# CDtoolX, a downloadable software package for processing and analyses of circular dichroism spectroscopic data

**DOI:** 10.1002/pro.3474

**Published:** 2018-10-18

**Authors:** Andrew J. Miles, B. A. Wallace

**Affiliations:** ^1^ Institute of Structural and Molecular Biology, Birkbeck College University of London London United Kingdom

**Keywords:** circular dichroism spectroscopy, data analyses, data processing, synchrotron radiation circular dichroism, data archiving, calibration

## Abstract

Circular dichroism (CD) spectroscopy is a highly used method for the examination and characterization of proteins, including, amongst other features, their secondary and tertiary structures, thermal stability, comparisons of wildtype and mutant proteins, and monitoring the binding of small molecules, folding/unfolding pathways, and formation of macromolecular complexes. This article describes CDtoolX, a new, user‐friendly, free‐to‐download‐and‐use software program that enables processing, displaying, archiving, calibrating, comparisons, and analyses of CD and synchrotron radiation circular dichroism spectroscopic data.

## Introduction

Circular dichroism (CD) spectroscopy is a highly used method for biophysical characterizations of a wide range of biological molecules, including proteins, nucleic acids, peptides, and sugars. Both lab‐based CD instruments and instruments which use synchrotron radiation as their light sources (SRCDs)^1^ produce information on optical features (mostly in the ultraviolet or vacuum ultraviolet wavelength ranges) which can provide conformational information.

Lab‐based CD instruments from different manufacturers all produce their own types of output in different (and sometimes proprietary) formats, which make comparisons of data sets produced on different instruments challenging and time consuming. In 2004, we created a downloadable software package (CDtool)^2^ for processing and analysis of CD spectroscopic data with inputs in a range of different formats and units, and which would output results in a common “.gen” format. It has been utilized by more than 700 registered users over the past 14 years.

Fortunately, unlike commercial CD instruments, the usage of different formats has not been as common on SRCD instruments, since most of these were developed after the introduction of the CDtool .gen format and so nearly all have this as their main or optional output/input format.^3^

CDtool software has been freely available to registered users. It was developed for Windows systems, and throughout many upgrades of the Windows operating systems (and Windows emulators, which have enabled use on other operating systems such as MACs), it has remained stable and usable. This is quite a long lifetime for a downloadable software package. However, large changes associated with the developments of Windows 8 and then Windows 10 operating systems rendered it generally unusable, whilst the user community was still keen on having access to a version for these different platforms. Hence, we have developed CDtoolX, a downloadable software package with most of the previous functions retained or upgraded and a number of additional ones added; it has been primarily designed for use on Windows 10 (hence the designation “X” in the software name) machines, but is also usable on Windows 7 (and below) platforms. It also runs on Mac (OS X) and Linux platforms that have Windows 10 emulators installed. CDtoolX includes a wide range of functions for displaying, processing, analyzing, and archiving CD and SRCD data. This version along with sample test data, and instructions for installation, is freely downloadable without registration. The only requirement for the user is that they cite this paper if they use the software for data processing and/or analyses.

CDtool has been widely used as a processing and analysis tool in CD and SRCD studies on diverse samples, including proteins,^4^ nucleic acids^5^ and even chiral small molecules,^6^ as well as for examining samples in different physical forms such as monolayers,^7^ fibers,^8^ emulsions,^9^ and oriented samples.^10^ Some recent examples of its use in different applications include: protein folding/unfolding,^11^ thermal stability profiles,^12^ comparisons of wild type and mutant proteins,^13^ monitoring continuous flow dynamics measurements,^14^ identifying similarities in protein evolutionary relationships,^15^ comparisons of new spectra with downloaded Protein Circular Dichroism Data Bank (PCDDB) components,^16^ demonstrations of fidelity of folding of expressed proteins,^17^ instrumentation comparisons and calibrations,^18^ as well as “on‐the‐fly” displays, data processing and analyses at SRCD beamlines.^19^ All of these types of studies are still compatible with CDtoolX.

## Results

### General functions and features

**Figure 1:**
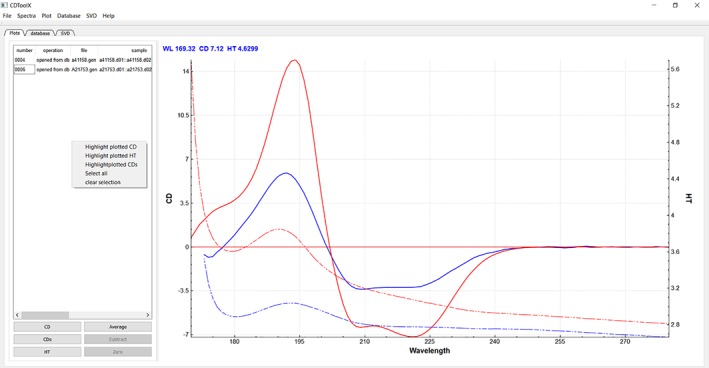
Image of the “Plot” tab page, showing the menu and tab function positions and the commands accessible from this tab, including the main spectral display. Listings of files in use are on the left hand side of the panel, as are the other function buttons described in the text. The CD (solid lines) and HT (dashed lines) plots are located to the right of the panel, with the associated HT spectrum displayed in the same color as its cognate CD spectrum. In this example, the red spectrum corresponds to the protein hemoglobin, and the blue spectrum is of alkaline phosphatase. Both spectra were downloaded from the PCDDB^22^ (codes CD0000037000 and CD0000002000), respectively, and are components of the SP175 reference data base.^27^

The main window (Fig. 1) has a menu bar at the top, with six drop‐down menus. The “File” menu has functions which enable the user to upload input files and save processed files to a selected directory. The “Plot” tab to the left of the plot window (under the tab buttons) lists the file names in use (and if they have already been processed, the component files that were used to create the file are displayed). The spectra are displayed in the plot window (right panel). Different files can be turned on and off in the plot window by selecting the appropriate file in the table and clicking the CD, CDS (for smoothed CD^20^), and HT (for high tension, a measurement that is called high voltage in some instruments and is related to the (unpolarized) absorbance of the sample^4^). Each spectrum is displayed in a different color, with the cognate CD (solid line) and HT (dashed line) spectra for the same sample depicted in the same color. Files can be averaged, subtracted or zeroed after highlighting their names, by clicking the boxes at the bottom of the left hand panel. Downloading and processing can be done on multiple files/data sets at the same time.

The “Spectra” menu contains functions which enable the user to apply a range of processes to the files selected in the File table (names highlighted). The third drop‐down menu, “Plot,” enables the user to change the display functions for the plotted spectra. This is augmented by a pop‐up menu invoked by right clicking the plot window or directly selected plots. The “Database” and “SVD” menus enable selection of other archiving and analysis functions described below. The “Help” menu leads to an “About” page with details for citation and usage of CDtoolX, as well as an extensive set of “Help” pages that form the user manual.

Below the menus are three tabs. Clicking the first tab labeled “Plot” displays the plotting window. Clicking the second tab labeled “Database” enables access to the user‐created archived database and its functions and clicking the third tab labelled “SVD” enables access to the component analysis functions.

### Input formats

Input files must be in ASCII format. Files which can be opened at time of this writing include those from different instruments and their different software versions and different SRCD beamlines, to wit: Aviv Biomedical v2.76 and v3.09, Jasco.txt files, Applied Photophysics Chirascan .cdnn format, ANKA beamline CD12, ISA Beamlines CD1, and UV1, Soleil beamline DISCO, and BSRF Beamline UV1 (SRCD 4B8 BSRF format). In addition, the generic .gen type format files produced by the original CDtool program and a simple two‐column (wavelength, CD value) format can be read. The .gen format is also available as a download format type for entries from the PCDDB files.^21^, ^22^

### Output formats

Output in the .gen format includes a “Headers” section for metadata, including processing procedures applied, and a summary of spectral data collection information. This section can be edited by the user. The data section that follows is arranged in five columns: wavelength, raw CD data, HT signal, CD data that has been smoothed with the Savitsky‐Golay function,^20^ and if the data is the average of more than one spectrum, the standard deviation between averaged spectra (otherwise the fifth column is filled with zeros). The output files are suitable for direct input into the DichroWeb (and other secondary structure analysis) websites^23^ as well as for deposition into the PCDDB. Graphical outputs of displays can be downloaded in .jpg format.

### Display

In the “Plot” tab, smoothed and unsmoothed CD plots and HT plots can be made visible (or hidden) by a toggling system in the lower left hand panel. Zooming is achieved using the mouse wheel and the plot can be dragged by holding down the left mouse button in the plotting display. This can be applied to either the X‐ or Y‐ or both axes. Either the CD or the HT plots can be zoomed (by selecting the appropriate (left or right) axis on the plot). Error bars can be displayed and hidden with a choice of sigma values and wavelength intervals by selecting the plot, then right clicking on the averaged spectrum plot and selecting “show error bars” from the pop‐up menu.

### Data processing

All processing functions can be performed on single or multiple files. Averaging, baseline subtraction and zeroing are performed using the buttons on the lower right hand panel of the Plot window. There is also a separate function accessible in the “Spectra” menu for subtracting a single baseline from multiple sample files. Alternatively, there are keyboard commands (described in the “Help” pages) to speed up the process. For example, “ctrl + A” will calculate the average of any highlighted spectra. The “Spectra” menu also contains functions for conversion to units of delta epsilon (DE) (the standard unit used in many publications to compare proteins of different molecular weights) or mean residue ellipticity (MRE) units from mdeg units (the standard instrument measurement units), following input by the user of the protein concentration, protein molecular weight and the sample cell pathlength. In addition, direct shape comparisons are enabled by using the “scale to values” function. Post‐data collection calibration procedures^24^ can also be applied via this menu (see below).

### Archiving

**Figure 2:**
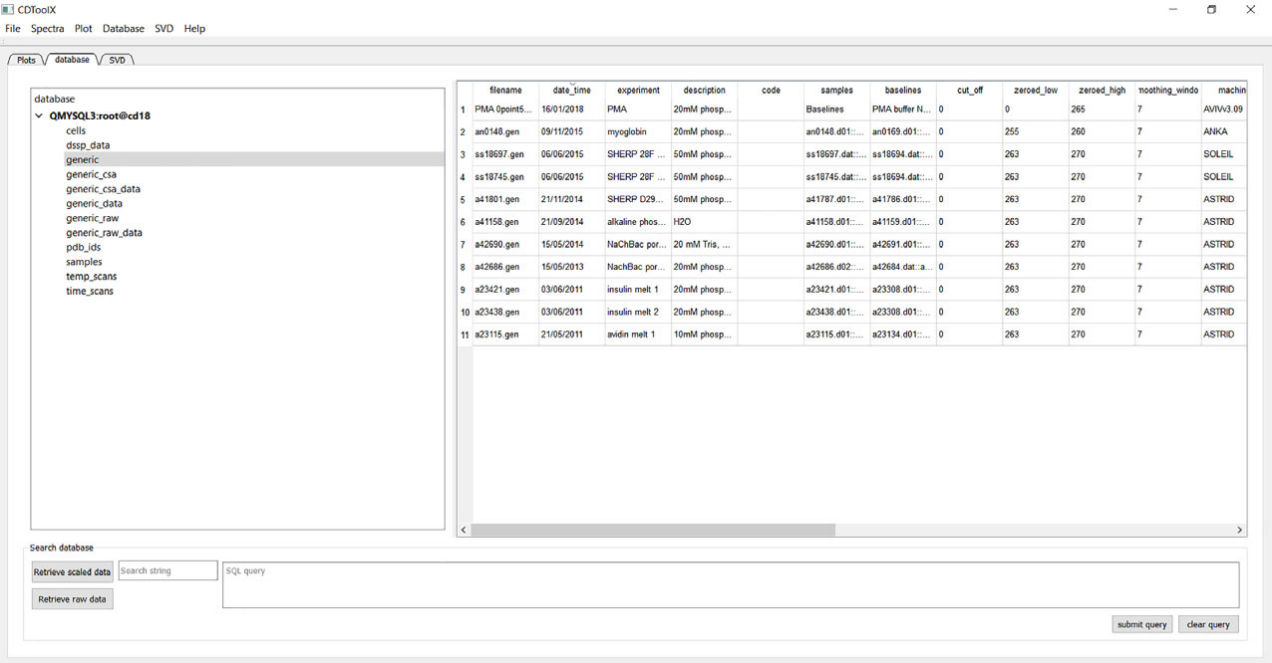
Image of the “Database” tab page, showing the database selected (grey overlay, left‐hand panel) and the entries and classes of information stored (in the table in the left panel), and the search function (bottom panel).

The menu function called “Database” accesses the archiving functions (Fig. 2). Both raw and processed spectra can be saved in a MySQL database. Once the user has obtained an open access version of MySQL, the database can be setup using files downloaded with CDtoolX. The setup for the database includes several entries in each table as a demonstration of how it can be populated. Raw or processed data can be retrieved from the database. The database can be searched either using a character string (lower left panel) or a MySqL query (lower right panel). Alternatively, data can be saved (either single or multiple files) as ASCII files, making them accessible to most spread sheet applications. Extensive descriptions of how the database can be set up and accessed are included in the “Help” menu.

### Analytical tools

**Figure 3:**
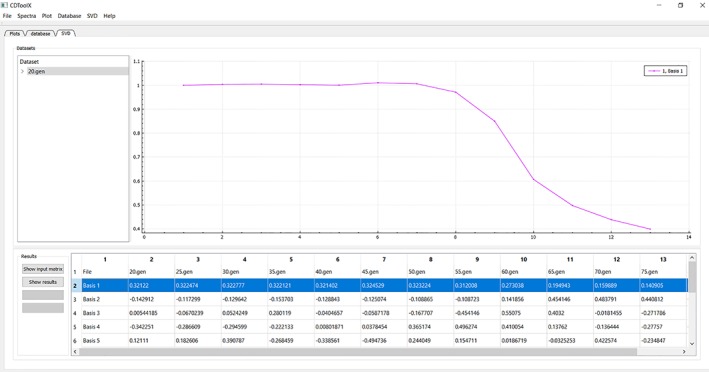
An example of an analysis using the “SVD” tab function. The name of the component files of the datasets used are in the upper left hand panel, with the right hand upper panel displaying the plot derived for the highlighted data set (lower right panel, blue overlay). The data used in these plots are from reference 12.

The third tab in the left panel enables access to functions for performing singular value deconvolution (SVD) analyses to identify component contributions, e.g., in thermal melt experiments^12^ (Fig. 3). To use this, the user needs to first upload all of the individual component spectra into the “Files” area by selecting them in the “Plot” tab list of files and choosing ‘create dataset’ from the SVD menu. The initial (unsmoothed) dataset is then displayed in a table, which can be saved as a .csv file. Rows, which correspond to a single wavelength representing, for example, the change in CD signal with temperature, can be previewed in the plot window or the data copied by right clicking the row and choosing the appropriate command from the menu that appears. The plot can be normalized (each data point divided by the first data point) to make direct comparisons possible, by selecting the plotted curve, then right clicking and choosing “Normalize plot” from the pop‐up menu that appears. The dataset, selected in the left hand file table (Fig. 2, dataset highlighted) can be edited (via a right click pop‐up menu) to remove wavelengths with compromised data; for example, all data points at wavelengths below where the HT values for that data set exceed the maximum HT cutoff value determined for the CD instrument which produced the data.^25^ The SVD analysis is performed by clicking the “Show Results” button on the left, whereupon the table in the lower panel displays the component contributions in each file. Each column represents one spectrum file and each row represents one principal component (eight are calculated, but the user must decide on the significance level and hence number of components that are relevant for their spectra). By right clicking on a row of values, a curve representing the contribution of that particular component to each spectrum can be previewed in the plot window or copied as described above. The entire table can be saved as a .csv file for plotting with, for example, standard spreadsheets, or other graphics software.

### Instruction manual/user help

The downloadable files accompanying the CDtoolX software include an extensive users’ manual. However, the software is designed to be very intuitive and user‐friendly so, in general, it can be used without reference to the manual. Users comments/questions can be addressed by emailing cdtools@mail.cryst.bbk.ac.uk.

## Discussion

CDtoolX offers a simple transparent method of data processing, enabling the user to identify inconsistencies in data due to, for example, non‐equilibration of the sample, which may manifest as spectral differences between repeat scans. Concentration errors can be resolved by scaling^26^ and conversion to DE. The HT signal can be used as a diagnostic tool for problems such as light scattering, bubble formation in temperature scans, and precise data cutoff points (wavelengths at which too little light reaching the instrument detector can be measured – corresponding to the lowest wavelength where the data is valid^4^). Moreover, facile comparison between data collected on different instruments and conventional and SRCD beamlines can be made. CDtoolX allows for data modifications including magnitude calibration,^26^ conversion to DE or MRE units, and calibration in the y axes.

Data is saved in ACSII format and arranged so that it can be easily displayed in spreadsheets. Files include user‐defined metadata. Simple plots can be rendered for demonstration and SVD reports and plots can be saved as .jpg files. The program allows for data archiving in a user‐created database from which data can be retrieved at a later time, and porting to create entries in the PCDDB.^22^ SVD component analyses of datasets can enable, for example, analyses of protein stability, especially as part of thermal melt studies.^12^

## Materials and Methods

The program is written in C++. The user interface was created using the Qt 5.7 library and compiled with The Qt Creator IDE, 4.5.0. The plotting windows are rendered using QCustomPlot. SVD and smoothing algorithms use the standard C++ library.

CDtoolX will run on Windows XP, Windows 7 and Windows 10 or Linux or MaC OS X platforms with a Windows 10 emulator installed. There is a separate download for Windows 7 and earlier versions of Windows, due to their incompatibility with later database file functions.

### Accessibility

CDtoolX is available for downloading, free and without registration at http://www.cdtools.cryst.bbk.ac.uk.

Updates will be available from time to time. The database functions have been shown to work with MySQL community server version 5.7.2, using the template database. The MySQL server is downloadable from https://dev.mysql.com/downloads/windows/installer/5.7.html.

## Conflict of Interest

The authors declare no conflicts of interest.

## Brief Statement of Impact/Importance

Circular dichroism (CD) spectroscopy is a highly used method for the biophysical characterization of a biological molecules, particularly proteins, nucleic acids, peptides, and sugars. This article describes a downloadable user‐friendly software package for the processing, display, calibration, archiving, and analyses of CD and synchrotron radiation CD data. It replaces the 14 year old, highly used program CDtool, implementing new functions, and enabling use on modern computer operating systems.
